# Assessing UVA and Laser‐Induced Crosslinking via Brillouin Microscopy

**DOI:** 10.1002/jbio.202400401

**Published:** 2025-02-16

**Authors:** Christian A. Iriarte‐Valdez, Johannes Wenzel, Emilie Baron, Alexandra Y. Claus, Stefan Kalies, Karsten Sperlich, Oliver Stachs, Maria Leilani Torres‐Mapa, Alexander Heisterkamp

**Affiliations:** ^1^ Institute of Quantum Optics Leibniz University Hannover Hannover Germany; ^2^ Lower Saxony Center for Biomedical Engineering Implant Research and Development (NIFE) Hannover Germany; ^3^ Department of Ophthalmology Rostock University Medical Center Rostock Germany; ^4^ Department of Life, Light & Matter University of Rostock Rostock Germany

**Keywords:** Brillouin microscopy, collagen type 1, cornea, femtosecond crosslinking, keratectasia, UVA crosslinking

## Abstract

Keratoconus and other corneal ectatic disorders involve the degradation of collagen fibers, which compromises the corneal biomechanical properties. Ultraviolet‐A (UVA) crosslinking has emerged as the primary treatment to slow down collagen degradation. This treatment is limited in both penetration depth and spatial precision, potentially leading to unwanted side effects. This study compares the changes in biomechanical properties of corneas crosslinked with UVA irradiation and a near‐infrared femtosecond laser, using Brillouin microscopy. The biomechanical properties of the crosslinked regions were mapped in terms of Brillouin frequency shift in three dimensions. UVA crosslinking showed an average increase in Brillouin frequency shift of ~100 MHz. We demonstrate targeted spatial and axial corneal femtosecond crosslinking, with similar Brillouin frequency shift values to UVA in crosslinked regions.

## Graphical abstract

UVA crosslinking is the primary treatment to combat keratoconus. Nevertheless, unwanted side effects and limitations due to nonselective irradiation lead to the investigation of alternative irradiation treatments. This study shows similar changes in the Brillouin frequency shift of corneas crosslinked with UVA irradiation and a NIR femtosecond laser.

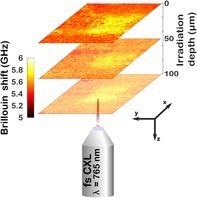

## 1 Introduction

Corneal ectatic disorders, such as keratoconus or iatrogenic cases, are noninflammatory eye conditions characterized by chronic biomechanical failure [1]. These disorders result from progressive weakening of collagen bonds in the corneal stroma, leading to deterioration of the primary corneal properties: stiffness and stability [2, 3, 4]. Patients with such disorders may suffer from severe astigmatism even at a young age, which leads to reduced quality of life [2, 4, 5].

Corneal crosslinking (CXL) is the only medically approved treatment that fundamentally treats the weakening of corneal tissue [6]. The treatment involves the interaction of a riboflavin solution with ultraviolet‐A (UVA) light irradiation at a wavelength of 365 nm. This combination induces the generation of reactive oxygen species and promotes the creation of new crosslinks between collagen fibers in the cornea which improves the corneal stiffness [7, 8]. While improving the biomechanical properties of the cornea, the treatment also aims to preserve corneal transparency, restrict the irradiated region on the corneal tissue, and reduce the treatment time [9]. To limit potential side effects and complications, numerous protocols have been developed. For example, accelerated crosslinking techniques have been applied based on the principle of photochemical reciprocity [10]. These protocols primarily reduce irradiation time by increasing UVA irradiation power, while maintaining the standard energy density of 5.4 J/cm^2^ [10, 11, 12]. However, evidence shows that cellular damage still occurs with accelerated protocols [13]. The need for a de‐epithelization (epi‐off) step before CXL also causes inflammation in some patients, which further adds to potential side effects [13, 14]. Customized corneal crosslinking has been developed to target a specific weak region in the cornea located in the keratoconic apex [15]. In this technique, the weakened region is crosslinked with a higher UVA energy density compared to surrounding areas. The method reduces the epithelial debridement area and concentrates the UVA irradiation profile to a smaller region which promotes faster healing [15, 16]. Preliminary results of customized corneal crosslinking have shown a stronger corneal flattening effect compared to the standard procedure but further studies and controls are necessary [15, 16, 17]. In general, studies have shown that UVA CXL results in a visible demarcation line, which separates the crosslinked from the unaffected region, where cellular changes and corneal structural alterations are visible (depths ~250 to 350 μm) [11, 14, 18]. Therefore, treatment of patients with thin corneas (< 400 μm) via UVA CXL is not recommended due to the potential damage to deeper tissue layers [13, 14, 19, 20].

Recent studies have proposed femtosecond crosslinking (fs CXL) as a promising alternative to UVA CXL [21, 22, 23, 24, 25, 26, 27]. Fs CXL is based on the multiphoton absorption process [26], which allows for spatially precise targeted crosslinking in the cornea and may reduce the risk of damaging the corneal endothelium or unwanted irradiation of healthy regions [26, 27]. Kwok et al. [21], implemented fs CXL on riboflavin‐treated bovine corneas (epi‐off) using a fs laser operated at a wavelength of 810 nm, 80 MHz repetition rate, and pulse width of 150 fs [21]. In comparison, fs CXL was performed on ex vivo porcine and in vivo rabbit corneas using a femtosecond laser operated at a wavelength of 1060 nm, repetition rates of 52 and 80 MHz, and pulse widths ~100 and 140 fs, without the addition of riboflavin wherein CXL was attributed to reactive oxygen species generated by low‐density plasma formation [22, 24]. Recently, fs CXL was performed on ex vivo human corneal stroma lenticules soaked in riboflavin using a chirped amplification laser system with a center wavelength of 800 nm, a repetition rate of 1 kHz, and varying pulse width between 60 and 120 fs [26, 27]. In contrast to UVA CXL, fs CXL is still considered to be in its early stages of research and neither a standardized protocol nor technical requirements have been fully established. Furthermore, due to different techniques used to assess fs CXL, the relative changes in biomechanical properties are difficult to compare among different studies (Table S1).

Several techniques have been implemented to evaluate corneal biomechanics after crosslinking. Both stress‐strain methods [28, 29] and atomic force microscopy [30] have been used to measure Young's modulus and empirically correlate the CXL treatment to the stiffening of the cornea, but are not suitable for in vivo measurements. Current non‐destructive techniques for clinical use in vivo include Ocular Response Analyzer (ORA, Reichert Technologies, Depew, New York) and Corneal Visualization Scheimpflug Technology (CorVis ST, OCULUS Inc., Arlington, Washington) [31, 32]. Both methods monitor corneal deformation and recovery in response to high‐speed air puff. Although used in clinical practice, these techniques still pose certain disadvantages, such as dependence on intraocular pressure, besides the lack of 3D information on the corneal biomechanics and quantitative information on the viscoelastic moduli [33]. Optical coherence elastography (OCE) is an emerging tool for in vivo characterization of three‐dimensional biomechanical properties [34, 35]. An intrinsic or externally applied perturbation to the tissue [36, 37, 38] can be used to calculate the tissue's viscoelastic properties [35, 39] by detecting the tissue response using optical coherence tomography [37, 38, 40, 41]. The perturbation can be static, leading to tissue displacement which is measured to calculate the tissue's stress‐strain response or dynamic wherein the propagation of elastic waves is analyzed [36, 37]. However, the need for a perturbation, whether external or physiological (e.g., *heartbeat*) [42, 43] makes this technique also highly dependent on biomechanical conditions, especially the intraocular pressure [35, 37, 39, 42, 43, 44], which has been shown to influence the measurements of the viscoelastic properties of the cornea [45].

Over the past decade, Brillouin microscopy has been applied as a nondestructive, optical method to quantify the biomechanics in the cornea [46]. Brillouin microscopy measures the inelastic scattering of light due to material‐intrinsic density fluctuations, which is dependent on the refractive index, density, and longitudinal modulus and can be quantified based on the Brillouin frequency shift (BFS) also called Brillouin shift [47]. This technique enables spatially resolved, depth‐dependent mapping of the mechanical tissue properties. Brillouin microscopy has been used to measure regional and depth‐dependent Brillouin frequency shift after crosslinking [21, 48], differentiate healthy and keratoconus corneas [49], as well as detect age‐related stiffening of lens [50]. Although both fs CXL [21] and UVA CXL [51, 52, 53] have been independently evaluated via Brillouin microscopy, a side‐by‐side comparison has not been reported in the literature.

In this study, we demonstrate three‐dimensional laser‐induced crosslinking on ex vivo porcine corneas using fs CXL. We perform fs CXL at a wavelength of 765 nm, optimal for two‐photon absorption of riboflavin [21, 54]. A customized Brillouin microscope is used to confirm targeted irradiation via fs CXL [51]. To provide evidence that the increase in Brillouin frequency shift truly corresponds to crosslinking, we measure UVA crosslinked cornea and pure collagen type I and further assess the enzymatic degradation rate of the crosslinked samples. Furthermore, we analyze the changes in Brillouin frequency shift before and after a standard crosslinking procedure. Thus, we contribute to ongoing efforts to standardize fs CXL protocol using Brillouin microscopy as an evaluation method. Overall, this study provides comprehensive information on the relative changes of the sample throughout the crosslinking process and compares the biomechanical changes that occur with UVA and fs crosslinked corneas in terms of Brillouin frequency shift.

## 2 Materials and Methods

The experiments were divided into two groups, first, collagen samples were prepared and analyzed under different conditions, namely, at different dextran concentrations and crosslinking procedures by varying the energy density of UVA irradiation. Second, crosslinking experiments on corneas were performed. Crosslinking in corneas was carried out both with UVA irradiation and with a near‐infrared femtosecond laser.

### 2.1 Preparation of Collagen Type I Samples

Collagen type I (Bovine collagen solution, 10 mg/mL, Fibricol) was used at 80% (v/v) concentration. Collagen was mixed with 10% Dulbecco's Phosphate‐Buffered Saline (DPBS, P04‐36500, PAN‐Biotech) 10×, 8% NaOH (0.1 M), and 2% distilled water. The final pH of the solution was adjusted to be pH ~ 7 and incubated for 60 min at 37°C.

### 2.2 Corneal Storage

Porcine corneas were used in this study. Eyes were received within 24 h *post‐mortem*. The corneal epithelium was carefully removed with a surgical scalpel, without damaging the stroma, and the area of the cornea was extracted from the ocular globus. The isolated corneas were stored in cornea medium at 4°C and used within 1 week. The cornea medium consists of cell medium (DMEM, high glucose, D5796, Sigma Aldrich, Germany), 6% dextran (Leuconostoc spp., Mr.: 450000–650 000, 31 392, Merck, Germany), 2% fetal bovine serum (FBS, S0615, Biochrom, Germany), 1% penicillin/streptomycin (P/S, P06–07050, PAN‐Biotech, Germany) and 20 mM HEPES [55, 56].

We observed that incubating the cornea in the cornea medium without dextran induces extensive swelling, increases the thickness, and reduces the optical transparency. The addition of dextran to the cell culture medium reduced and regulated the swelling and in effect, maintained the corneal thickness [55, 57, 58]. It has also been shown that incubation of cornea in this medium supplemented with dextran results in a higher number of viable endothelial cells [55] and allows for longer corneal storage [57].

### 2.3 Dehydration Studies with Dextran and Enzymatic Degradation Assay

Collagen type I samples were prepared to analyze their properties and behavior under the effect of varying dextran concentrations (10%, 15%, and 20%). Each collagen sample had a volume of ~500 μL and was sectioned into three similarly sized pieces using a surgical scalpel, aided by customized 3D‐printed molds to ensure uniform dimensions.

We measured the weight, refractive index, Brillouin frequency shift, and enzymatic resistance as a function of dextran concentration. To determine the hydration of the samples, collagen pieces were left in the different dextran concentrations at 4°C, and the weight of the samples was recorded over time (0, 1, 2, 3, 4, 5, and 24 h). Changes in refractive index at 589 nm were measured using a refractometer (Abbe AR4, KRÜSS, Germany). Enzymatic resistance measurements were performed using 500 μL collagenase I at a concentration of 1 mg/mL (SCR103, Merck, Germany) at 30°C. O‐Phthaldialdehyde (OPA, P0532, Merck, Germany) assay was employed to quantify the peptide concentration in the degraded material [59]. 5 μL of the degraded material (supernatant) was combined with 50 μL of OPA reagent. Supernatants of the samples were collected after 1, 2, 3, 6, and 24 h of degradation. The resulting fluorescence was detected using a microplate reader (Tecan Infinite 200 Pro, Tecan Austria, Austria) with an excitation wavelength of 340 nm and emission wavelength of 455 nm. The intensity of the fluorescence is proportional to the amount of degraded peptides, allowing for quantification of collagen breakdown over time. To determine the relationship between fluorescence level and peptide concentration, a calibration curve using bovine serum albumin (BSA, A94, Merck, Germany) was prepared to determine the peptide concentration in the supernatant. BSA concentrations ranging from 1 to 50 mg/mL were used to confirm the linearity of the fitted curve.

### 2.4 Crosslinking Protocols

**FIGURE 1:**
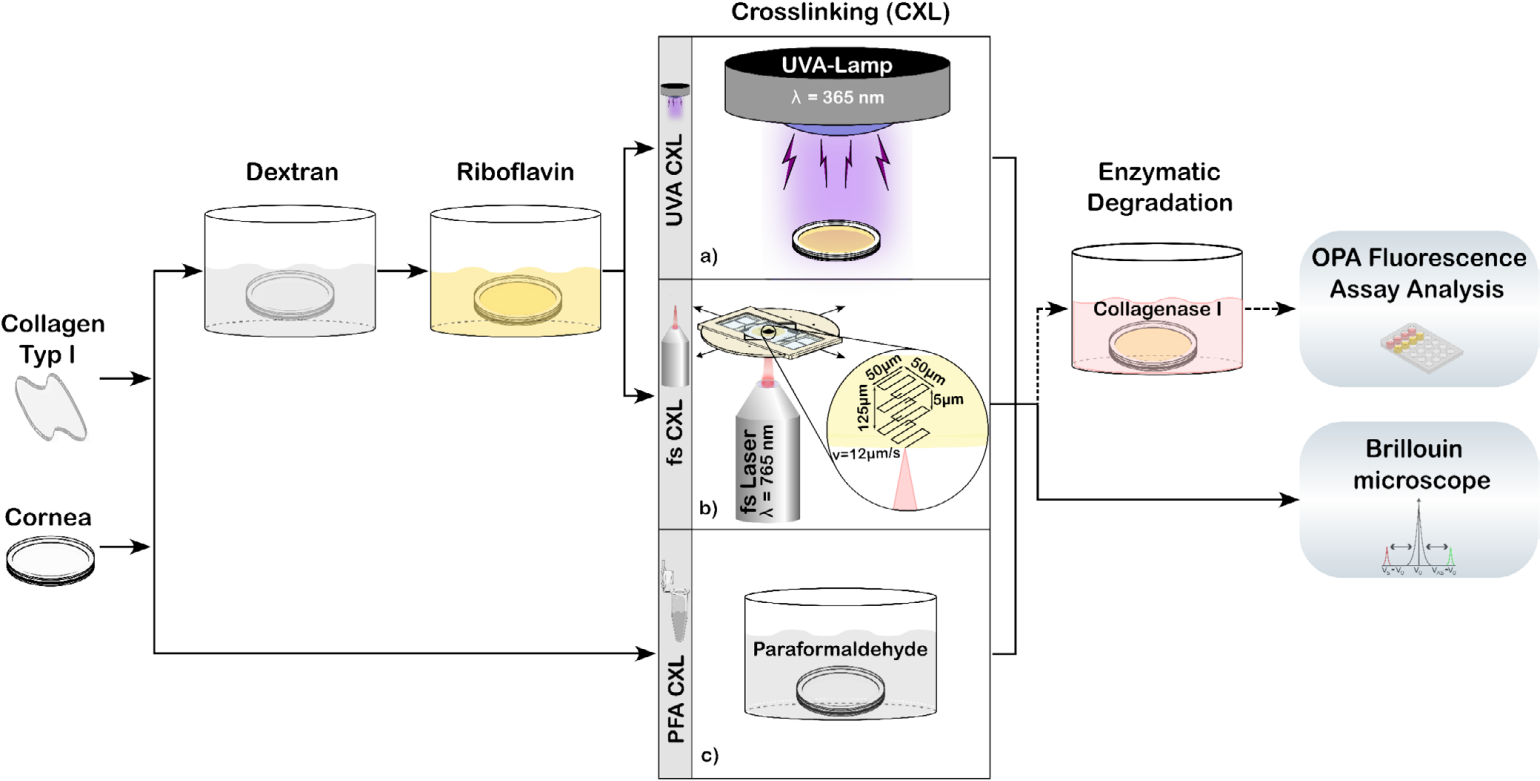
Schematic diagram of the crosslinking procedure for collagen type I samples and porcine corneas. Samples were first incubated in dextran solution, followed by the addition of riboflavin. Crosslinking was performed using one of the following methods: (a) UVA and (b) fs‐laser irradiation and (c) chemical crosslinking through paraformaldehyde. The analysis was performed either via enzymatic degradation assay or Brillouin microscopy.

Figure 1 shows a schematic diagram illustrating the different crosslinking protocols for collagen and cornea samples, along with the various analytical methods employed. Both collagen and corneas were crosslinked using UVA irradiation and paraformaldehyde (PFA, 0335.2, Roth, Germany).

Collagen samples were cut into six similarly sized pieces using a custom 3D‐printed mold to ensure sample uniformity. To ensure that the sample dehydration is the same as prior crosslinking, the samples were immersed in 15% dextran overnight. One of the pieces was maintained in dextran throughout (dextran control). Four pieces were immersed in 0.1% riboflavin/10% dextran solution for 30 min (Riboflavin 5′‐monophosphate sodium salt hydrate, F1392, Merck, Germany). One of these samples was maintained in riboflavin prior measurements (riboflavin control) and the three pieces were irradiated with UVA light (λ = 365 ± 10 nm) at different energy densities, 2.7, 5.4, and 10.8 J/cm^2^ [60], using a UVA lamp (IROC UV‐XTTM 2000, IROC Innocross, Switzerland). The last piece was crosslinked with PFA. Crosslinking with PFA was performed by immersing the sample in 4% PFA for 10 min, followed by rinsing and gentle shaking of the sample in DPBS for 1 h, with medium change every 15 min.

The corneas were treated following the same protocol as collagen samples, but cut into four pieces instead: dextran control, riboflavin control, UVA crosslinked with an energy density of 5.4 J/cm^2^ [60, 61] and crosslinked using PFA [59].

A total of 12 collagen samples and 11 corneas were UVA crosslinked. They were either analyzed using Brillouin microscopy or their resistance to enzymatic degradation was measured using OPA fluorescence assay.

Fs CXL was performed on four corneas. Samples were incubated overnight in 15% dextran. Before crosslinking, corneas were immersed in 0.1% riboflavin/10% dextran for 30 min. The corneas were irradiated using a Ti: sapphire femtosecond pulsed laser (Chameleon Vision II, Coherent, USA) with a pulse rate of 140 fs and repetition rate of 80 MHz at a wavelength of 765 nm. The power at the laser output was controlled by using a half‐wave plate combined with a polarizing beam splitter and set to a fixed laser power of 180 mW, with a corresponding pulse energy of ~2.25 nJ and a peak power of ~16 kW. A customized 3‐axis, computer‐controlled stage positioned the samples through the laser beam, with a scanning speed of 12 μm/s according to the selected pattern and size. The beam was focused to a spot size of ~1 μm on the anterior of the de‐epithelialized cornea through an objective (Plan‐Apochromat, 63×, NA 1.0, Zeiss, Germany). The irradiation pattern was a cube consisting of a square pattern with dimensions of 50 × 50 μm^2^, composed of parallel lines with a distance of 3 μm. The distance between irradiated squares in the axial direction was 5 μm, reaching ~125 μm in tissue depth. For fs crosslinked samples, only Brillouin microscopy was performed, since the size of the crosslinked areas was too small to perform the enzymatic degradation analysis.

During the riboflavin absorption and crosslinking procedure, samples were kept in the dark throughout the experiments. During fs CXL treatment and Brillouin microscopy, the samples were held in a customized 3D‐printed holder. The samples were mounted between two coverslips, which ensured homogeneity in hydration during the entire process, as well as flattened the samples for uniform irradiation.

Wilcoxon signed‐rank test was implemented for statistical significance tests, taking into account the distribution of our results and the number of samples to be analyzed by comparing the controls to each condition.

### 2.5 Brillouin Microscope

A custom‐built Brillouin microscope reported in our previous study [51] was employed to measure the Brillouin frequency shift under various crosslinking conditions. The system utilized a tunable diode laser (DL Pro 780, Toptica Photonics AG, Germany) at a wavelength of 780.24 nm. A half‐wave plate combined with a polarizing beam splitter regulated the laser power and ensured linear polarization. The beam was then directed to an etalon (OP‐7423‐6743‐2, Light Machinery Inc., Canada), which suppressed amplified spontaneous emission background noise. A pair of computer‐controlled scanning mirrors (GVS012/M, Thorlabs Inc., USA), combined with a scan lens (LSM03‐VIS, Thorlabs Inc., USA) and a tube lens, facilitated sample scanning over a specified area, ensuring uniform distance between points. A quarter‐wave plate polarized the incident beam circularly before it entered the objective, and converted the polarization of the backscattered light to linear, oriented at 90° with respect to the incident beam's initial polarization. The laser beam, with an approximate power of 8 mW, was focused into the sample using a 10× MPlan Apo objective with an NA 0.28 (Edmund Optics Inc., USA) with a lateral and axial resolution of 1 and 12.5 μm, respectively.

The scattered beam was directed to an Rb‐vapor cell, maintained at 70°C to suppress the Rayleigh peak. The beam enters a VIPA spectrometer, which includes a cylindrical lens to focus the signal into a VIPA (OP‐6721‐3371‐4 Light Machinery Inc., Canada). A convex lens collected the spectrometer's output and directed it to the sCMOS camera (Andor Zyla 4.2 Plus, Oxford Instruments plc., UK).

The system has to be calibrated, to avoid systematic errors that could arise due to day‐to‐day differences in external conditions. The Brillouin frequency shift (ν_B_) for water with a value of 5.12 GHz, was calculated using the equation (1) [51].

(1) $$\nu_{B}=\frac{2n}{\lambda }\cdot V\cdot \sin\left(\frac{\theta }{2}\right)$$

where *n* = 1.333 is the refractive index of water, *V* ≈ 1498 m/s is the phase velocity of sound for a temperature *T* ≈ 25°C, λ is the laser wavelength, and scattering angle, $\theta =180^{{}^{\circ}}$ for backscattering geometry. This calculated value serves as a calibration reference. Comparing the Stokes and anti‐Stokes pixel distance in water to the collagen and cornea samples enables the determination of their corresponding Brillouin frequency shift. This calibration was repeated before every sample measurement [51].

## 3 Results

### 3.1 Effect of Collagen Type I Hydration State on Its Biomechanical Properties

**FIGURE 2:**
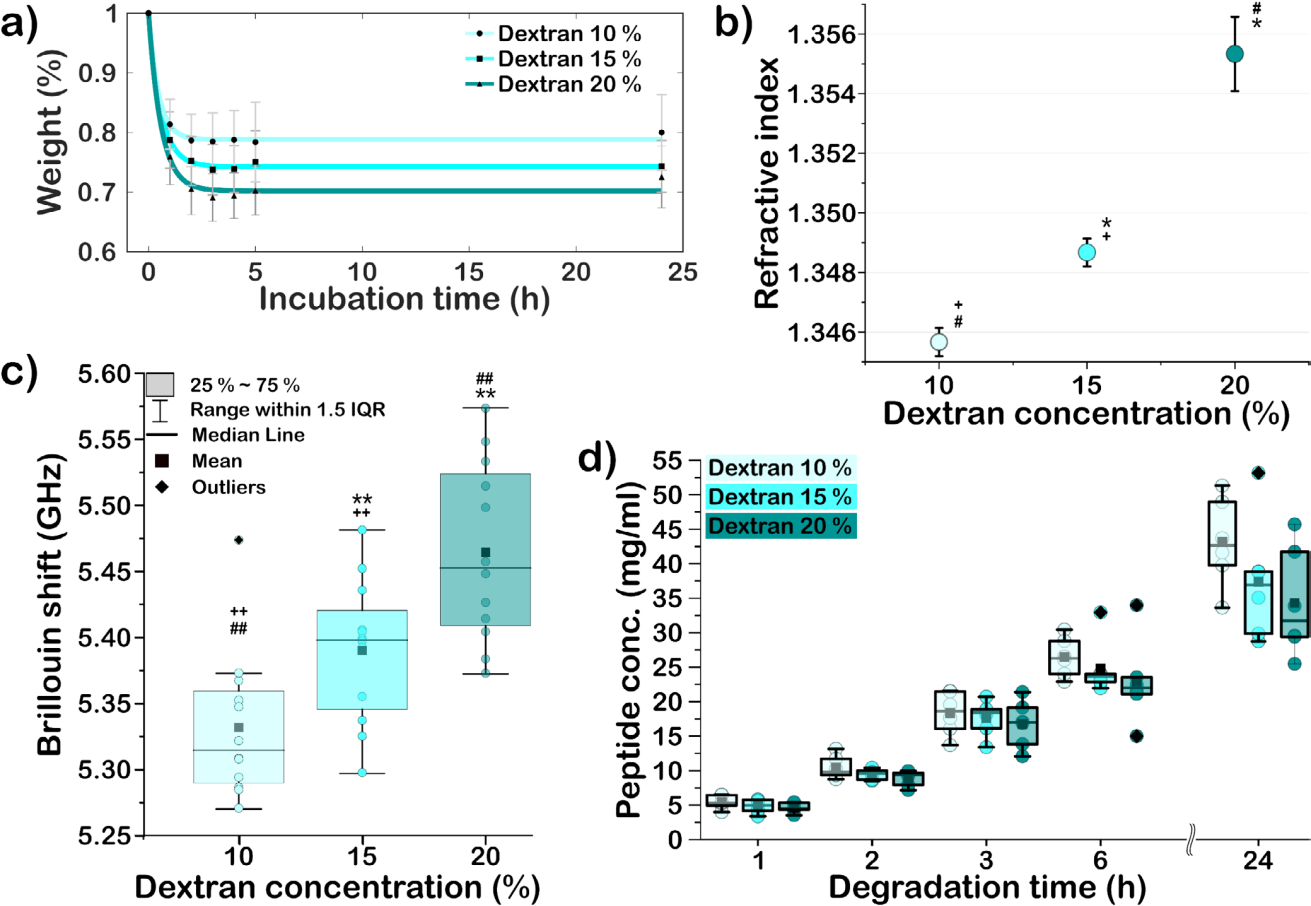
Effect of dextran concentration on collagen type I samples. (a) Sample weight measurements at different time points and different dextran concentrations (*n* = 6). (b) Refractive index as a function of dextran concentration (*n* = 6). (c) Brillouin shift as a function of dextran concentration, measured by Brillouin microscopy (*n* = 6). (d) Peptide concentration after enzymatic degradation of collagen samples over time (*n* = 6). The comparison to 10%, 15%, and 20% dextran is denoted by *, # +, respectively (*p < 0.05, **p < 0.01, ***p < 0.001).

Since the biomechanical properties of the cornea are dependent on its hydration state [62, 63], we investigated the dehydration effect of dextran on the Brillouin frequency shift of collagen type I. Dextran acts as an osmotic agent, regulating and maintaining an optimal and constant level of corneal hydration throughout the treatment, which is essential for the efficacy and safety of CXL [64, 65]. Previous studies have observed that decreasing hydration increases corneal tissue stiffness [66]. Given that collagen type I is the predominant component in the cornea and responsible for its stiffness, we investigated the effect of varying dextran concentrations of 10%, 15%, and 20% on collagen samples (Figure 2). By measuring the refractive index and weight changes, we confirmed how the hydration state of the samples varies as a function of dextran concentration. We measured the collagen biomechanical properties by performing Brillouin microscopy and compared the results by evaluating the amount of degraded peptide using an enzymatic degradation assay. The degradation assay reports the resistance of the material degradation to collagenase exposure due to biochemical changes in the collagen's tertiary structure and reduced access to cleavage sites [59]. Increased enzymatic resistance is correlated to enhanced material tensile strength and mechanical integrity [67].

Figure 2a shows the changes in the collagen weight over time for different dextran concentrations. The weight of the collagen samples soaked in 10%, 15%, and 20% dextran was reduced to 80%, 75%, and 70% of their original weight, respectively. Dehydration of collagen samples reached their maximum for all dextran concentrations after 3 h, with the most significant dehydration occurring within the first hour.

The dehydration was further confirmed by refractive index measurements, as shown in Figure 2b. The measured refractive indices increased with higher dextran concentrations and deviated further from that of water, with the highest refractive index of ≈1.355 measured for collagen samples immersed in 20% dextran concentration.

The Brillouin frequency shift of the collagen type I samples immersed at varying dextran concentrations was measured using the Brillouin microscope. Measurements were taken at a single plane, ~ 50 μm from the surface for all samples. The samples exhibited an increase in Brillouin frequency shift with increasing dextran concentrations, as shown in Figure 2c. Based on the statistical tests, the medians of the samples were significantly different from each other (*p* < 0.05). The effect of dextran on collagen samples and their biomechanical properties was further analyzed by enzymatic degradation assay. Figure 2d shows slightly slower degradation rates for samples incubated at higher dextran concentrations, however, the differences were not statistically significant. The results support previous Brillouin measurements on the cornea, where they observed that Brillouin frequency shift increases with greater sample dehydration [68]. The Brillouin frequency shift varies directly to the radical of the longitudinal modulus, which is inversely correlated to the overall tissue compressibility [69]. Since tissue compressibility can be approximated as a linear combination of the solid and fluid components of the tissue, Brillouin microscopy will be sensitive to the relative changes in the material's hydration state and solid constituent [62]. Our results show that in the absence of changes in the intra and intermolecular bonds of the sample, hydration of collagen type I correlates inversely to Brillouin frequency shift [62, 69].

### 3.2 UVA Crosslinking of Collagen Type I

Based on our hydration experiments on collagen type I, we designed the crosslinking protocol to achieve highly reliable Brillouin measurements and analysis. To ensure that the samples will have a similar initial hydration state, the collagen samples were kept overnight in 15% dextran before CXL. Instead of immersing the samples in 0.1% riboflavin/20% dextran, we used 0.1% riboflavin/10% dextran for optimal diffusion of riboflavin prior to irradiation. Collagen samples were irradiated at various UVA energy densities to clearly distinguish the effect of crosslinking in terms of Brillouin frequency shift.

**FIGURE 3:**
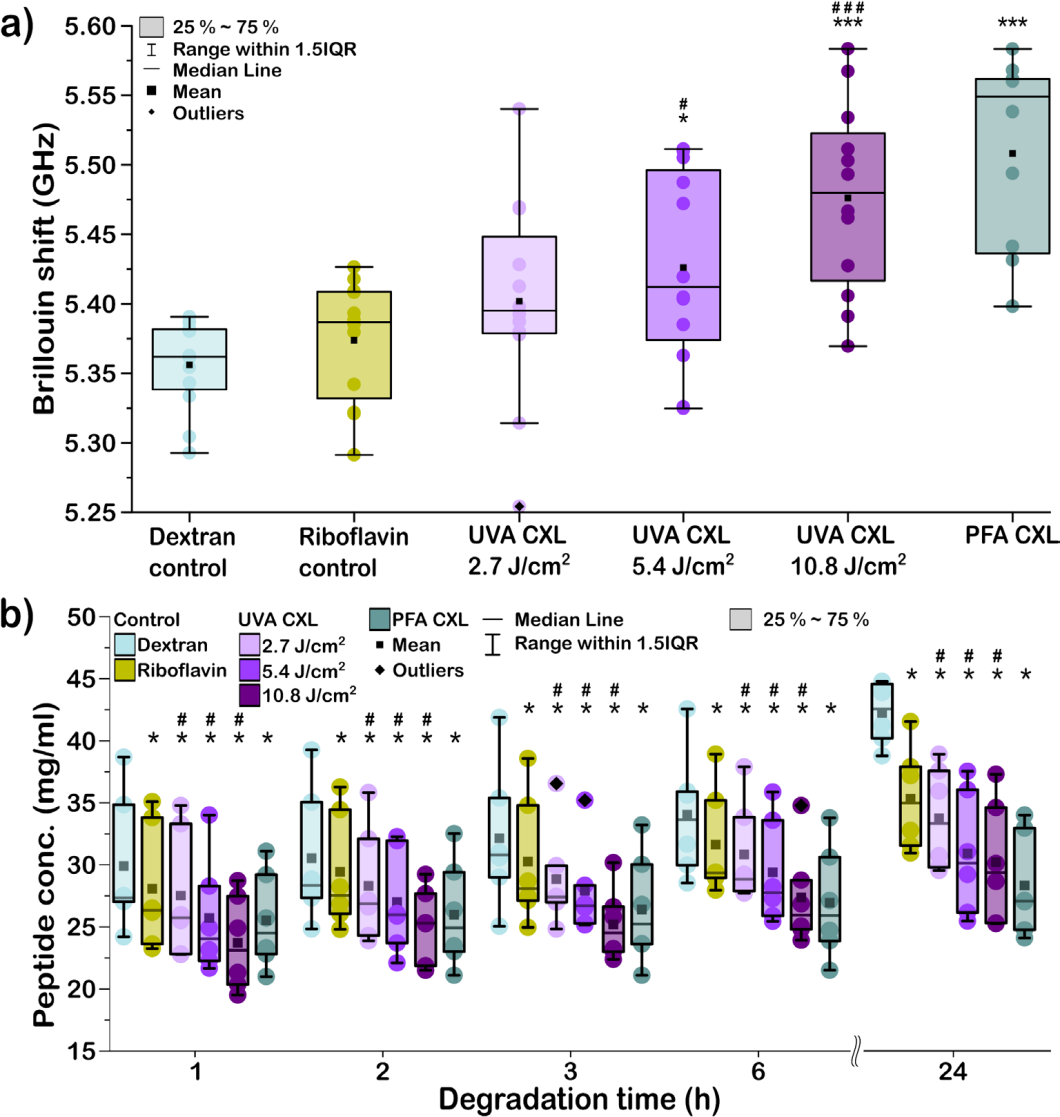
Comparison of crosslinking methods on collagen type I evaluated using enzymatic degradation and Brillouin microscopy. (a) Brillouin shifts for different crosslinking methods in comparison to control (nontreated) and riboflavin‐immersed samples (*n* = 6). (b) Peptide concentration as a function of degradation time for different crosslinking conditions (*n* = 6). Comparison to dextran and riboflavin control is denoted by symbols *, #, respectively (*p < 0.05, ***p < 0.001).

Figure 3a presents the Brillouin frequency shift measured on collagen controls, crosslinked via UVA irradiation at various energy densities of 2.7, 5.4, and 10.8 J/cm^2^ and PFA. The results of the enzymatic degradation assay are shown in Figure 3b. Previous research [59] indicated that chemical crosslinkers, such as paraformaldehyde (PFA), exert a stronger effect than UVA irradiation. Consequently, the PFA crosslinking of collagen samples serves as a reference for the crosslinking process.

Both Brillouin microscopy and enzymatic degradation methods demonstrated a slight increase in biomechanical integrity following the application of riboflavin, although the increase in Brillouin frequency shift was not statistically significant. In contrast, the degradation assay showed a slower increase in peptide concentration over time, which is statistically valid (*p* < 0.05, compared to dextran control). Our study also revealed an enhanced material strength correlating with rising UVA energy density. Similar to riboflavin, the measured Brillouin frequency shift at 2.7 J/cm^2^ did not show statistical significance compared to controls. However, the amount of degraded material for UVA crosslinked at 2.7 J/cm^2^ compared to the dextran control sample and riboflavin control samples was statistically significant. Higher energy densities and PFA crosslinking exhibited the most substantial increase in mechanical strength. Brillouin measurements using a standard protocol (5.4 J/cm^2^) were statistically significant compared with control and riboflavin samples (Figure 3a). Furthermore, both 10.8 J/cm^2^ and PFA crosslinking demonstrated the highest statistical significance (*p* < 0.001).

The enzymatic degradation results illustrate that UVA irradiation at 10.8 and 5.4 J/cm^2^ has a more pronounced effect than PFA during the initial hours of degradation (Figure 3b). The samples irradiated with 10.8 J/cm^2^ consistently displayed higher Brillouin frequency shifts and slower degradation rates than samples treated with 5.4 J/cm^2^, despite that crosslinking using both energy densities exhibited similar behavior throughout the degradation period. After 24 h of exposure to collagenase I, PFA demonstrated a more profound crosslinking effect than UVA irradiation. Our results show that the increase in Brillouin frequency shift is correlated to enhanced crosslinks in collagen type I fibers as validated by the enzymatic degradation assay.

### 3.3 3D Brillouin Mapping of UVA and PFA Crosslinked Cornea

We performed similar measurements on corneas at varying depths using Brillouin microscopy and validated the results using an enzymatic degradation assay. Corneas were irradiated with an energy density of 5.4 J/cm^2^. Brillouin maps were taken at different tissue depths, close to the surface, 50 and 100 μm from the surface. The relative tissue depth was measured based on the first plane where a Brillouin signal was detected.

**FIGURE 4:**
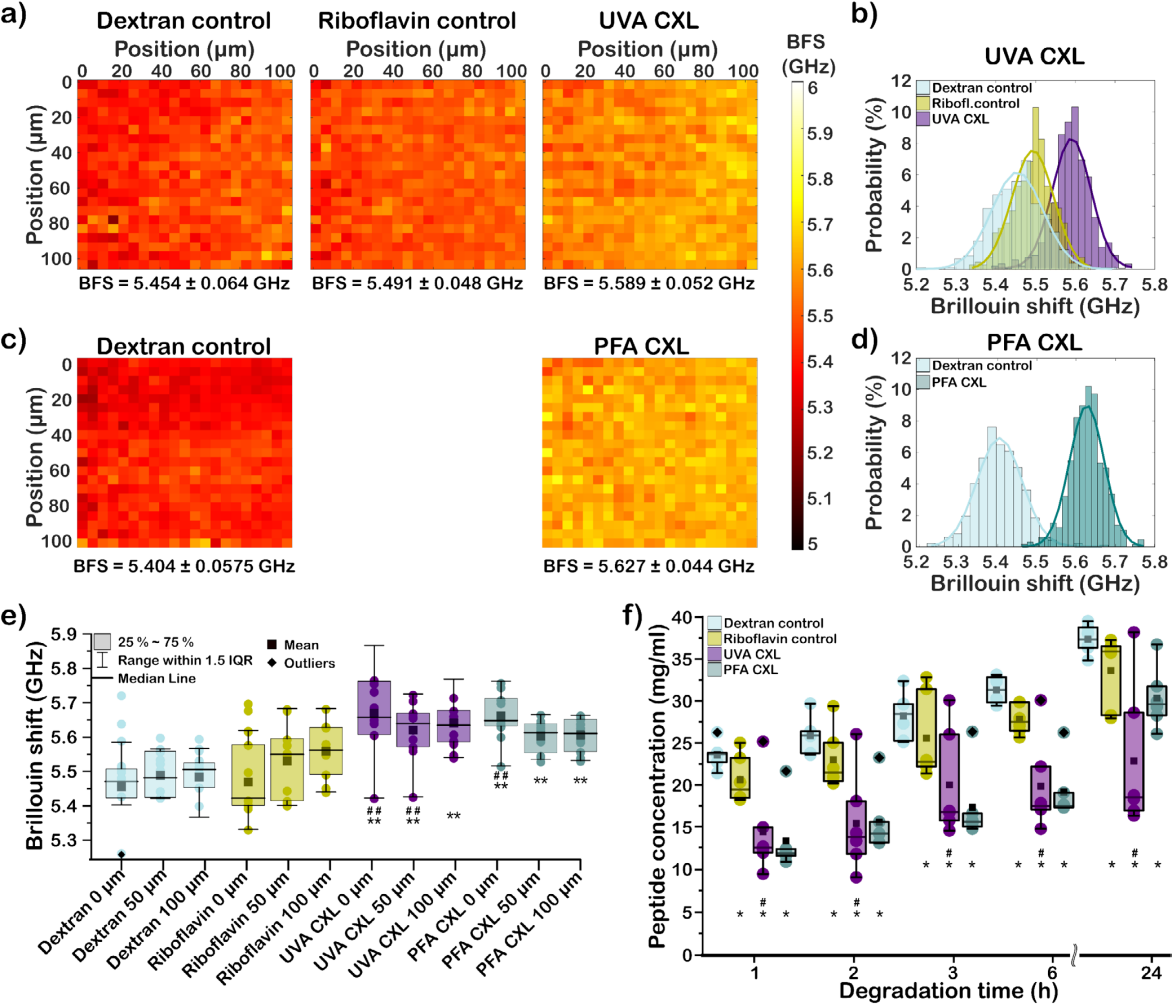
Comparison of UVA CXL and PFA CXL on the porcine cornea using Brillouin microscopy and enzymatic resistance analysis. (a) Brillouin maps showing changes throughout the UVA CXL process, from control sample to post‐UVA irradiation. (b) Respective probability distributions of Brillouin frequency shift (BFS) for all measured points across the UVA CXL stages. (c) Brillouin maps of the PFA CXL process before and after cross‐linking. (d) Probability distribution of Brillouin frequency shift for PFA CXL. (e) Comparison of Brillouin shifts between dextran and riboflavin controls, as well as UVA and PFA CXL, measured up to ~100 μm depth (*n* = 5). (f) Enzymatic degradation over time for each crosslinking method is presented as peptide concentration (*n* = 6). Comparison to dextran and riboflavin control is denoted by symbols *, #, respectively (*p < 0.05, **p < 0.01).

Figure 4a shows representative Brillouin maps of dextran control, riboflavin control, and UVA CXL taken at a tissue depth of 100 μm. The average Brillouin frequency shift for each measurement is shown below the respective Brillouin map.

A slight increase in average Brillouin frequency shift ~40 MHz is visible after the application of riboflavin and an increase of more than 100 MHz is measured after UVA CXL. Figure 4b plots the distribution of the measured Brillouin frequency shift for each condition. Dextran control showed the broadest distribution curve, followed by riboflavin control with the narrowest exhibited by UVA CXL. Brillouin frequency shifts for UVA CXL resulted in the least FWHM of ~113 MHz and the highest average Brillouin frequency shift of ~5.58 GHz. For comparison, we also present a representative measurement of PFA CXL compared to a corresponding dextran control at 100 μm depth (Figure 4c). We measured a difference > 200 MHz between PFA‐crosslinked and control samples. Similar to UVA CXL, a narrower FWHM, and higher average Brillouin frequency shift was measured for PFA CXL compared to the dextran control (Figure 4d).

Figure 4e presents the Brillouin frequency shift at different tissue depths of UVA‐ and PFA‐crosslinked corneas. The relative change in Brillouin frequency shift of crosslinked samples compared to control decreased with increasing tissue depths which is consistent with the study of Scarcelli et al. [48]. For UVA CXL, a small increase in Brillouin frequency shift after application of riboflavin is visible. The results were compared with the enzymatic degradation assay (Figure 4f). Riboflavin‐treated corneas showed a small increase in biomechanical integrity. Corneas treated with UVA and PFA crosslinking exhibited lower degraded peptide concentration compared to riboflavin and dextran controls. In contrast to UVA crosslinked samples, PFA CXL showed less variation in the measured peptide concentration. At 24 h, the concentration of degraded peptides is higher for PFA compared to UVA crosslinked corneas which could be attributed to the short treatment time and limited PFA absorption in corneas. Consistent with the results for collagen type I, both UVA and PFA CXL in the cornea demonstrate a significant increase in Brillouin frequency shift which corresponds to slower enzymatic degradation rates.

### 3.4 Selective Spatial and Axial Corneal Crosslinking Using Femtosecond Laser Irradiation

**FIGURE 5:**
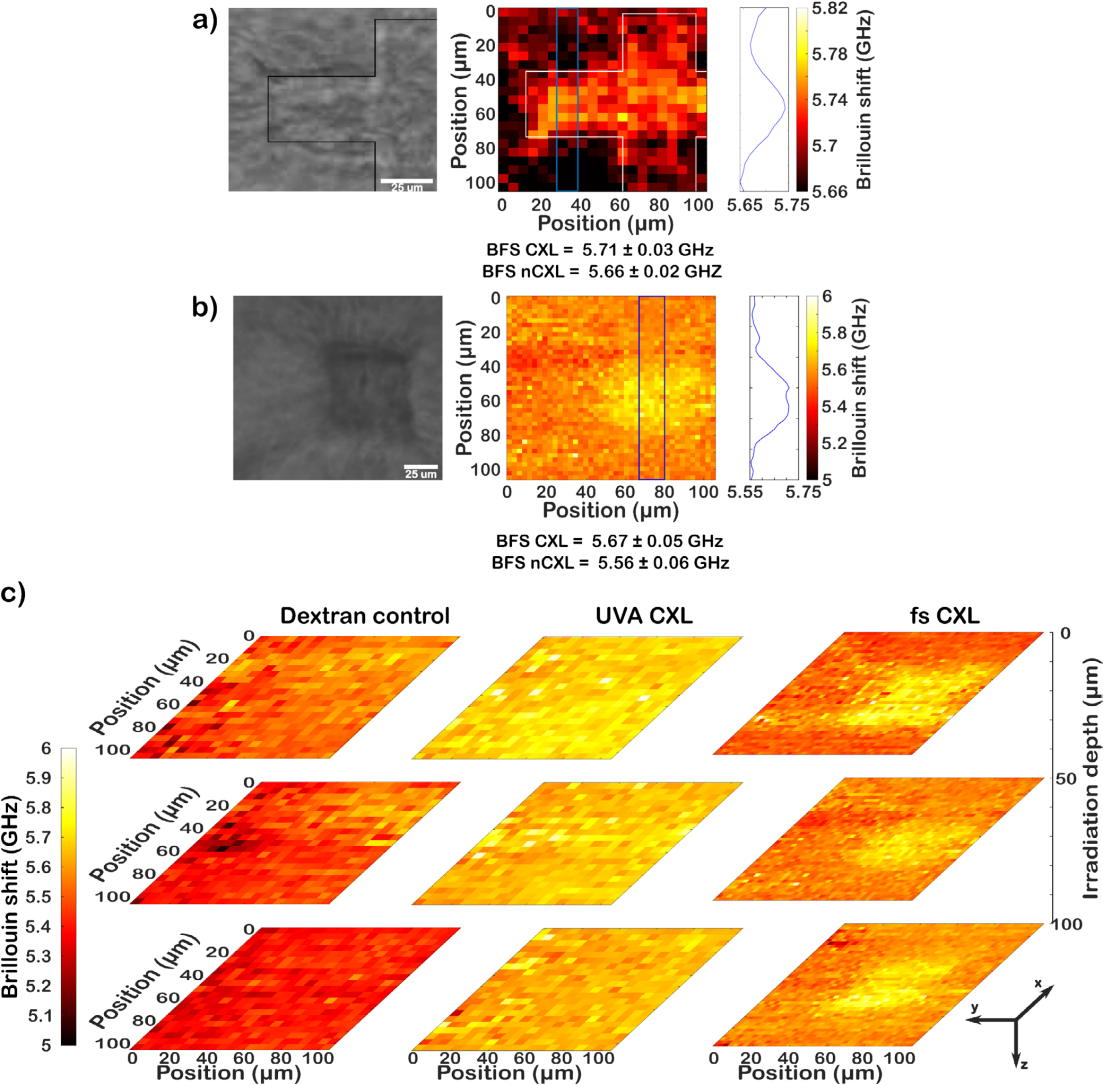
Femtosecond laser‐assisted corneal crosslinking analysis. Targeted crosslinking shows different irradiance patterns with (a) a cross and (b) a square pattern. Left: Brightfield microscope images of irradiated areas. Middle: Corresponding Brillouin microscopy maps. Right: The line profile of Brillouin shifts in the blue‐marked areas of the Brillouin maps. The average Brillouin frequency shift (BFS) for the corresponding area is stated below the Brillouin maps. (c) Comparison of Brillouin frequency shifts for three different tissue depths of non‐crosslinked corneas (left), UVA‐crosslinked corneas (middle), and fs CXL‐treated corneas (right).

As a final experiment, we performed spatially selective near‐infrared (NIR) fs CXL by irradiating defined patterns in porcine corneas. Figure 5a,b, illustrates the targeted area where laser‐induced CXL was performed. The irradiated patterns were cross and square, with a corresponding relative change of ~0.9% and 1.85% in Brillouin frequency shift compared to surrounding nonirradiated areas, respectively. Figure 5c shows a representative comparison of the Brillouin maps measured for dextran control, UVA CXL, and spatially selective fs CXL for different tissue depths. Close to the surface, fs CXL showed a relative change of ~2.84%, while at 50 and 100 μm deeper in tissue, the relative change achieved was ~2.70% and 1.96%, compared to nonirradiated regions, respectively. Notably, fs CXL demonstrates an increase in Brillouin frequency shift, spatially localized within the predefined irradiated volume.

**FIGURE 6:**
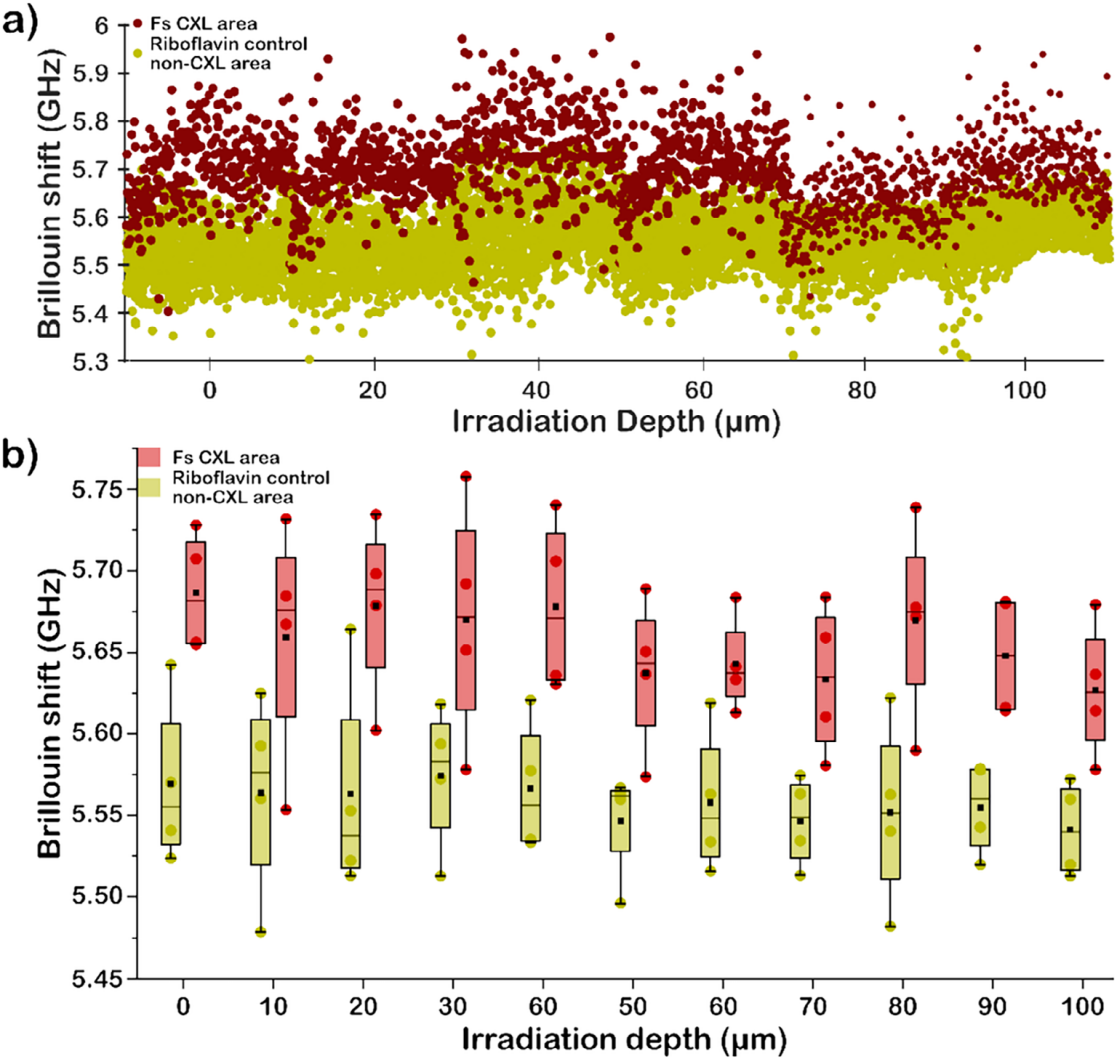
Depth‐dependent analysis of Brillouin frequency shift in crosslinked and non‐crosslinked corneal regions. (a) Representative scatter plot of individual Brillouin shift measurements at various depths, comparing non‐crosslinked (yellow) and crosslinked (red) areas. (b) Comparison of average Brillouin shift values at different depths between crosslinked and non‐crosslinked regions (*n* = 4).

The average differences for a total of four samples treated with fs CXL are shown in Figure 6. Figure 6a shows a representative measurement of all Brillouin frequency shifts measured in one cornea as a function of tissue depth. The Brillouin frequency shifts for the non‐crosslinked measured points were lower than the crosslinked areas.

Figure 6b shows a comparison of the average Brillouin frequency shifts between crosslinked and non‐crosslinked areas for all irradiated corneas. The measured crosslinked values, in general, have a difference of ~100 MHz compared to the non‐crosslinked regions, which represents an average relative change of ~1.8%. Similar Brillouin frequency shift values for the measured tissue depths were obtained for both fs CXL and UVA irradiation. In contrast to UVA CXL wherein UVA penetration depth in a scattering tissue is limited, the Brillouin frequency shift as a function of tissue depth remained constant due to the deeper penetration depth using a femtosecond laser operated in the near‐infrared regime. Overall, the results demonstrate that fs CXL exhibits a high degree of spatial and depth crosslinking control within the cornea tissue which can be confirmed and visualized by 3D mapping using Brillouin microscopy.

## 4 Discussion

In ophthalmology research, a tool that allows for 3D volumetric mapping of biomechanics in the eye would be extremely valuable to assess therapeutic intervention post‐treatment and progression of disease. Brillouin microscopy shows promise as a noninvasive method in quantifying the viscoelastic properties of biological tissues and cells in a noncontact manner [51, 52, 70]. This technique offers higher precision compared to OCE [35, 40] and provides corneal measurements with minimal influence from intraocular pressure [35, 40, 45, 66, 71]. Combining the results of Brillouin microscopy with OCE could enhance the understanding of the biomechanical properties of tissues since these methods provide complementary viscoelastic moduli that are independent of each other [35]. OCE allows the measurement of shear and Young's moduli, while the Brillouin frequency shift (ν_B_) can be correlated to the real part of the tissue's longitudinal modulus (M'), as described by Equation (2).

(2) $$M^{\prime }=\frac{\rho {\nu_{B}}^{2}\;\lambda^{2}}{4\eta^{2}\;}$$

where ρ is the tissue density, λ is the wavelength and η is the refractive index of the tissue [66]. However, the ratio $\rho /\eta^{2}$ requires independent measurements and can be highly variable depending on environmental conditions [45, 62, 72]. Therefore, we opted to directly report the Brillouin frequency shift values and compare the relative change of these values for each condition. As recommended, an alternative would be to present Brillouin measurements in terms of a dimensionless parameter, Brillouin elastic contrast, $\bar{\upsilon_{B}}=\left(\frac{\upsilon_{B}}{\upsilon_{B}^{w}}\right)–1$, where $\upsilon_{B}^{w}$ is the Brillouin frequency shift of water, to account for the differences in laser wavelength used in different Brillouin microscopy systems [73]. Several studies have demonstrated a clear correlation between tissue biomechanical properties to Brillouin frequency shift in various biological materials such as zebrafish [74], whole mouse embryos [70] or even distinguishing different cellular compartments based on their mechanical properties [75].

The biophysical interpretation of Brillouin measurements in hydrated biopolymers and biological tissue has been subject to debate [76, 77] whether the technique probes the material viscoelastic properties rather than their water content. In high water content hydrogels, (> 90%) Brillouin measurements are affected predominantly by hydration rather than material biomechanical properties [76]. Recent results have demonstrated though, that Brillouin spectroscopy can effectively characterize sol‐gel transitions in gelatin, a denatured form of collagen [78]. At high polymer volume fractions, water may be considered bound to collagen molecules with even more enhanced coupling at an increasingly dehydrated (glass transition) state [78, 79]. Studies in cornea showed that changes in Brillouin frequency shift due to hydration occur due to the contribution of water to the overall tissue compressibility [62]. Furthermore, for crosslinked corneas under controlled hydration conditions, the Brillouin shifts account for the changes in the solid part of the biomechanics of the tissue [69]. These studies demonstrate that Brillouin frequency shift is sensitive to the viscoelastic nature of the biopolymer sample indicative of its biomechanics and material state [77, 78].

We evaluated the changes in biomechanical properties for collagen type I samples with varying hydration states by measuring the Brillouin frequency shift and the concentration of degraded peptides. Both enzymatic degradation assay and Brillouin microscopy are excellent methods to evaluate material biomechanical properties changes [67, 69, 80]. Brillouin microscopy has been demonstrated to be sensitive to the changes in both the fluid and solid part of tissue compressibility, thereby both factors can influence the measured Brillouin frequency shift [62, 69]. Meanwhile, enzymatic degradation assay reports the biochemical changes and corresponding enhanced mechanical resistance to enzyme degradation due to additional bonds formed between collagen fibers [67]. We observed the dependence of the Brillouin frequency shift on hydration using pure collagen type I immersed in diverse dextran concentrations. Lower dextran concentrations induced higher hydration levels in samples, resulting in a decrease in Brillouin frequency shift and vice versa. The enzymatic degradation of non‐crosslinked collagen samples did not show a statistically significant difference in the concentration of degraded peptides between collagen samples at different hydration states, since no known changes in their biochemical structure are expected when collagen is simply immersed in dextran.

We performed UVA and PFA crosslinking on both collagen type I and cornea samples and validated the increase in Brillouin frequency shifts using an enzymatic degradation assay. In crosslinked collagen type I, a proportional relationship between irradiation energy density and Brillouin frequency shift was observed. These results support previous studies that reported an increase in corneal tissue stiffness as a function of UVA energy densities [81]. In general, an increase in Brillouin frequency shift is visible when comparing the changes in crosslinking states to control or riboflavin‐treated samples. We also demonstrated that Brillouin frequency shift changes after UVA and PFA CXL correspond to a slower rate of enzymatic degradation, which could indicate stronger crosslinks between collagen fibrils in the corneal stroma. A reduced enzymatic degradation rate has been shown to indicate stronger bonds after CXL between lamellae in corneal tissue as confirmed by histological examination and implied an increase in mechanical stiffening [26, 82]. Biochemical alterations in collagen may include enhanced concentrations of primary and secondary amines as well as methylene and methyl groups [83]. UVA CXL has also been shown to create higher molecular weight polymer chains [84] and build crosslinks between the collagen side chains' amino terminals and the extracellular matrix [84]. Additionally, reduced interfibrillar spacing [84] and voids have been reported [26], which altogether may explain the reduced degradation rate observed on both crosslinked collagen and cornea samples.

The effect of riboflavin was also observed and analyzed. A small increase in Brillouin frequency shift was measured in collagen samples as well as in corneal tissue independent of tissue depth. This observation was also made by Melcher et al. [85] using surface‐enhanced Raman scattering analysis. This implies that the mere application of riboflavin in ambient lighting can already induce the crosslinking process in collagenous tissue. Although the changes in Brillouin frequency shift between riboflavin and dextran control were not statistically significant, a clear increase in enzymatic resistance for riboflavin‐treated samples was observed. Irradiation with UVA light of the riboflavin‐treated corneas reduced the degradation rate and showed a significant change in Brillouin frequency shift.

Lastly, we evaluated the changes in cornea tissue biomechanics using fs CXL by comparing the Brillouin frequency shift changes after UVA CXL and fs CXL. 3D Brillouin frequency shift maps of the femtosecond laser irradiated porcine tissues showed a highly precise region with increased Brillouin frequency shift. For UVA crosslinking at 5.4 J/cm^2^, measurements on collagen samples showed an average difference of 65 MHz, and corneas exhibited an average difference of about 100 MHz between crosslinked and non‐crosslinked samples. The measured Brillouin frequency shift for UVA CXL showed a decrease as a function of tissue depth [48, 52]. In contrast, the Brillouin frequency shift for fs CXL remained constant in tissue depth. In a previous study, a change in Brillouin frequency shift ~30 MHz in femtosecond crosslinked cornea was reported for depths below 80 μm [21]. On average, similar average Brillouin frequency shifts were measured for both UVA and fs CXL which demonstrates the effectivity of fs irradiation for corneal crosslinking. Aside from enhancing the biomechanics of cornea, an additional advantage of fs laser irradiation is its ability to locally modify the refractive index of the sample, as demonstrated previously in glass [86] and hydrogels [87]. With more established parameters and protocols, it would be foreseeable that fs laser irradiation could also be used to treat a keratoconus patient's vision loss while at the same time improving the cornea's biomechanical properties. Overall, we demonstrated the potential of fs CXL for highly precise, spatially targeted crosslinking at depth, which overcomes some of the major limitations in UVA CXL such as limited UV light absorption in corneal tissue and reduced spatial targeting resolution.

## 5 Conclusions

In this study, we compared the changes in corneal crosslinking using UVA and laser‐induced CXL and evaluated the corresponding tissue changes using three‐dimensional Brillouin microscopy. We performed a careful comparison using consistent protocols in corneal handling and also confirmed the results on crosslinked collagen type I samples. Changes in Brillouin frequency shift, after localized near‐infrared femtosecond laser irradiation were comparable to changes seen before and after UVA CXL. UVA CXL induced a proportional increase in Brillouin frequency shift as a function of energy density. Fs CXL has shown a higher Brillouin frequency shift compared to non‐crosslinked regions and constant Brillouin frequency shift as a function of tissue depth. Thus, we demonstrated that fs CXL can induce a similar increase in Brillouin frequency shift and correspondingly biomechanical changes compared to UVA CXL but with distinct advantages on spatial precision in irradiation as well as maintained biomechanical properties within the irradiated region.

## Conflicts of Interest

The authors declare no conflicts of interest.

## Supporting information


**Data S1.**.

## Data Availability

The data that support the findings of this study are available from the corresponding author upon reasonable request.
