# Supplementary material for: Assessing UVA and Laser‐Induced Crosslinking via Brillouin Microscopy
Source: J Biophotonics. 2025 Feb 16;18(5):e202400401. doi: 10.1002/jbio.202400401 (PMC12022390; doi:10.1002/jbio.202400401)
Supplement: Supplementary file 1 — Data S1.. [file JBIO-18-e202400401-s001.docx]

**Assessing UVA and Laser-induced Crosslinking via Brillouin microscopy**

Christian A. Iriarte-Valdez^1,2^, Johannes Wenzel^1,2^, Emilie Baron^1,2^, Alexandra Y. Claus^1,2^, Stefan Kalies^1,2^, Karsten Sperlich^3,4^, Oliver Stachs^3,4^, Maria Leilani Torres‑Mapa^1,2,*^, Alexander Heisterkamp^1,2,*^

| **Source** | **Laser Laser Specification** | | | | **Treatment** | | | | | | **Analysis** | |
| --- | --- | --- | --- | --- | --- | --- | --- | --- | --- | --- | --- | --- |
|  | **Type** | **λ (nm)** | **Pulse width (fs)** | **Rep. Rate (MHz)** | **Irrad. Power (mW)** | **Pulse Ener. (nJ)** | **Scan speed (mm/s)** | **Obj. NA** | **Ribo-flavin** | **Sample** | **Method** | **General findings** |
| **Kwok *et al.* (2017); [16]** | Ti:Sapphire | 810 | 150 | 80 | 104 | 1.30 | 0.012 | 1 | yes | porcine corneas epi - off | Brillouin Microscopy | Increase in Brillouin shift (~5.5%) |
| **Wang *et al.* (2017); [17]** | Ti:Sapphire | 1060 | 140 | 80 | 60 | 0.75 | 30 | 0.6 | no | porcine eyes epi-on | Inflation test | Decrease in diopter/irradiated layer |
| **Shavkuta *et al. (2018*); [18]** | SHG Tema-100 (Er) | 525 | 200 | 70 | 100 | 1.43 | 500 | 0.1 | yes | Human cornea (*ex-vivo)* epi - off | Nanoindentation | Increase in elastic modulus |
| **Wang *et al.* (2018); [19]** | Ti:Sapphire | 1060 | 99 | 52 | 60 | 1.15 | 2.2 | 0.6 | no | porcine eyes (*ex-vivo*) rabbit eyes (*in-vivo*) epi-on | EPR spectroscopy | Decrease in diopter |
| **Bradford *et al.* (2019); [20]** | Laser amplifier Ti:Sapphire | 760 | 130 | 0.05 – 0.1 | 30 | 300 - 600 | 5 - 100 | 0.12 | yes | rabbit eyes (ex vivo) epi - off | Indentation Elasticity | Increase in Young’s modulus |
|  |  |  |  |  |  |  | 20 |  |  | rabbit eyes (*in-vivo)* epi - off |  |  |
| **Chang *et al.* (2022); [21]** | Ti:Sapphire | 800 | 120 | 0.001 | 4.5 | 4500 | 2 | 0.6 | yes | human corneas SMILE lenticule extracted epi - off | Stress - strain test | Increase in elastic modulus (~6 %) |
| **Cheng *et al.* (2023); [22]** | Ti:Sapphire | 800 | 60 | 0.001 | 2.3 | 2300 | 0.667 | 0.6 | yes | human corneas SMILE lenticule extracted epi - off | Stress - strain test | Increase in Young's modulus (~ 300%) |
|  |  |  |  |  |  |  | 1 |  |  |  |  |  |
|  |  |  |  |  | 4.5 | 4500 | 0.667 |  |  |  |  |  |
|  |  |  |  |  |  |  | 1 |  |  |  |  |  |
|  |  |  | 120 |  |  |  | 1.33 |  |  |  |  |  |
|  |  |  |  |  |  |  | 2 |  |  |  |  |  |
| **this study** | Ti:Sapphire | 765 | 140 | 80 | 180 | 2.25 | 0.012 | 1 | yes | porcine corneas epi - off | Brillouin Microscopy | Increase in Brillouin shift (~1.9%) |

Table 1. Overview of studies that demonstrated femtosecond crosslinking in cornea samples including laser specification, treatment and evaluation method.
